# Genetic inactivation and pharmacological blockade of sigma-1 receptors prevent paclitaxel-induced sensory-nerve mitochondrial abnormalities and neuropathic pain in mice

**DOI:** 10.1186/1744-8069-10-11

**Published:** 2014-02-11

**Authors:** Francisco R Nieto, Cruz M Cendán, Francisco J Cañizares, María A Cubero, José M Vela, Eduardo Fernández-Segura, José M Baeyens

**Affiliations:** 1 Department of Pharmacology, Biomedical Research Centre and Institute of Neuroscience, University of Granada, 18012 Granada, Spain; 2 Department of Histology, Biomedical Research Centre and Institute of Neuroscience, University of Granada, 18012 Granada, Spain; 3 Esteve, Drug Discovery and Preclinical Development, Parc Científic de Barcelona, Carrer Baldiri Reixac, 4-8, 08028 Barcelona, Spain; 4 Current address: Wolfson Centre for Age-Related Diseases, King’s College London, Wolfson Wing, Hodgkin Building, SE1 1UL London, UK

**Keywords:** Paclitaxel, Sigma-1 receptors, Chemotherapy-induced peripheral neuropathy, BD-1063, Mitochondria, Allodynia, Neuropathic pain, Sigma-1 receptor knockout mice

## Abstract

**Background:**

Paclitaxel, a widely-used antineoplastic drug, produces a painful peripheral neuropathy that in rodents is associated with peripheral-nerve mitochondrial alterations. The sigma-1 receptor (σ_1_R) is a ligand-regulated molecular chaperone involved in mitochondrial calcium homeostasis and pain hypersensitivity. This receptor plays a key role in paclitaxel-induced neuropathic pain, but it is not known whether it also modulates mitochondrial abnormalities.

In this study, we used a mouse model of paclitaxel-induced neuropathic pain to test the involvement of the σ_1_R in the mitochondrial abnormalities associated with paclitaxel, by using genetic (σ_1_R knockout mice) and pharmacological (σ_1_R antagonist) approaches.

**Results:**

Paclitaxel administration to wild-type (WT) mice produced cold- and mechanical-allodynia, and an increase in the frequency of swollen and vacuolated mitochondria in myelinated A-fibers, but not in C-fibers, of the saphenous nerve. Behavioral and mitochondrial alterations were marked at 10 days after paclitaxel-administration and had resolved at day 28. In contrast, paclitaxel treatment did not induce allodynia or mitochondrial abnormalities in σ_1_R knockout mice. Moreover, the prophylactic treatment of WT mice with BD-1063 also prevented the neuropathic pain and mitochondrial abnormalities induced by paclitaxel.

**Conclusions:**

These results suggest that activation of the σ_1_R is necessary for development of the sensory nerve mitochondrial damage and neuropathic pain produced by paclitaxel. Therefore, σ_1_R antagonists might have therapeutic value for the prevention of paclitaxel-induced neuropathy.

## Background

Paclitaxel is a first-line antitumor agent that frequently produces neuropathic pain, for which no treatment is available [1,2]. However, experimental models of paclitaxel-induced neuropathy are allowing the testing of novel treatments and/or the elucidation of pathophysiological mechanisms e.g., [3-6]. Emerging evidence suggests that paclitaxel-induced neuropathy is a consequence of toxic effects on mitochondria. In particular, an increased incidence of swollen and vacuolated axonal mitochondria in peripheral sensory fibers appears to be relevant [7,8]. This increase in atypical mitochondria has been attributed to the binding of paclitaxel to mitochondrial β-tubulin, which may produce Ca^2+^ release from mitochondria and dysregulated intracellular Ca^2+^ homeostasis [7,9]. In fact, paclitaxel-induced neuropathic pain in rodents is prevented and/or reversed by agents that either enhance mitochondrial function [3,8,10] or reduce intracellular Ca^2+^[4,9,11,12]. Alterations of mitochondrial function and/or intracellular Ca^2+^ levels may also contribute to other peripheral neuropathies [13].

The sigma-1 receptor (σ_1_R) has been identified as a ligand-regulated molecular chaperone [14] and proposed as a modulator of several receptors and ion channels [15]. Specifically, at mitochondrion-associated endoplasmic reticulum membrane (MAM) level, the σ_1_R chaperone modulates the intramitochondrial Ca^2+^ level and plays a key role in the control of intracellular Ca^2+^ homeostasis [16]. σ_1_Rs are highly expressed in the central and peripheral nervous system, including important areas for pain control [17-19], and the involvement of σ_1_R in pain modulation is well documented [20-26]. Accordingly, σ_1_Rs have been proposed as an emerging target for the treatment of neuropathic pain [27]. Our group recently reported that paclitaxel-induced pain is reduced in σ_1_ receptor knockout (σ_1_R-KO) mice and in wild-type (WT) mice treated with σ_1_R antagonists [28]. However, it is not known whether the beneficial effects of σ_1_R blockade on paclitaxel-induced neuropathic pain are associated with a reduction of the mitochondrial abnormalities induced by the antineoplastic.

In this study, we first compared the paclitaxel-induced changes in hind-paw pain perception (acetone and electronic Von Frey tests) and in saphenous nerve mitochondrial characteristics (transmission electron microscopy analysis) in WT mice. We then evaluated whether σ_1_R inhibition by treatment with a selective σ_1_R antagonist (BD-1063) or genetic inactivation (σ_1_R-KO mice) prevents the neuropathic pain behaviors and mitochondrial changes induced by paclitaxel. The present report shows that paclitaxel treatment induces pain behaviors in parallel with the occurrence of mitochondrial abnormalities in WT mice, and that pharmacologically- or genetically-induced σ_1_R blockade prevents both types of abnormality.

## Results

### Behavioral pain studies

**Figure 1:**
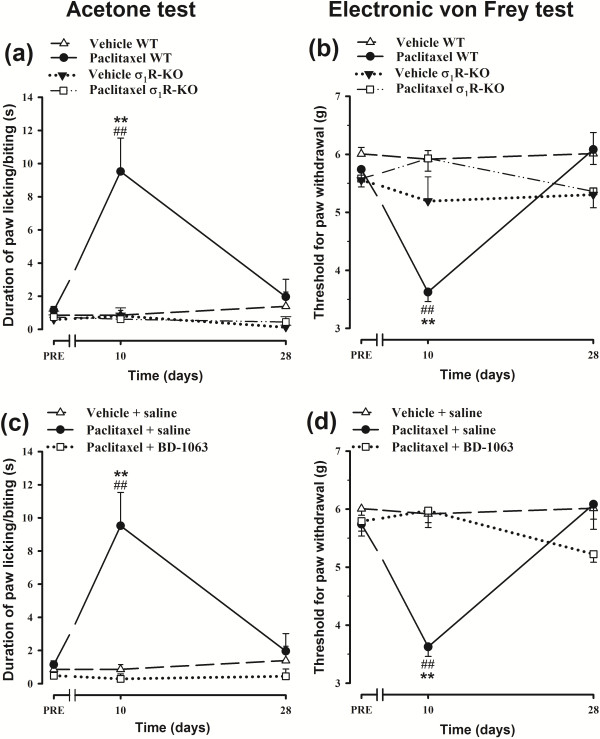
**Effects of σ**_**1**_**R blockade in paclitaxel-induced neuropathic pain behaviors.** Effect of paclitaxel + saline and paclitaxel-vehicle + saline on the duration of hind paw licking/biting in the acetone test **(a)** and on the threshold force for hind paw withdrawal in the electronic Von Frey test **(b)** in WT and σ_1_R-KO mice. Time-courses of the effect of paclitaxel + saline, paclitaxel + BD-1063 (32 mg/kg), and paclitaxel-vehicle + saline in the acetone test **(c)** and in the electronic Von Frey test **(d)** in WT mice. The animals were treated once daily from days 1 to 5 with an i.p. injection of paclitaxel (2 mg/kg) or its vehicle **(a,b)** and with an s.c. injection of BD-1063 or saline 30 min before each paclitaxel dose **(c,d)**. The response was recorded in each animal 3 days before (PRE) and on days 10 and 28 after the start of treatment. Each point and vertical line represents the mean ± SEM of the values obtained in 6–12 animals. Statistically significant differences between WT mice treated with paclitaxel + saline and the rest of the groups on the same day after treatment, **p < 0.01; and between the values on the pretreatment day and the days after treatment, ## *p* < 0.01 (two-way repeated measures ANOVA followed by Bonferroni test).

On the pretreatment day, WT and σ_1_R-KO mice showed a similar duration of acetone-induced paw licking/biting (Figure 1a) and a similar threshold force for paw withdrawal in the electronic Von Frey test (Figure 1b). Treatment with paclitaxel vehicle did not significantly modify the response of WT or σ_1_R-KO mice in any test at any measurement time point (Figure 1a and b). However, on day 10 after the first paclitaxel injection, WT mice showed a statistically significant increase in acetone-induced paw licking/biting duration (cold-allodynia) and a reduction in threshold force for paw withdrawal in the electronic Von Frey test (mechanical allodynia) (Figure 1a and b). On day 28 after the first paclitaxel dose, the response of WT animals had returned to normal values in both tests (Figure 1a and b). In contrast, the paclitaxel-treated σ_1_R-KO mice showed no sign of cold or mechanical allodynia at any time point (Figure 1a and b). Likewise, the s.c prophylactic administration of the selective σ_1_R antagonist BD-1063 (32 mg/kg) to WT animals, totally prevented the development of cold and mechanical allodynia (Figure 1c and d). Therefore, σ_1_R inhibition induced by genetic inactivation (σ_1_R-KO mice) or treatment with a selective σ_1_R antagonist (BD-1063) completely averted the neuropathic pain behavioral manifestations induced by paclitaxel administration.

All treated animals were in good general health at the end of the treatment and all gained weight normally (data not shown).

### Electron microscopy analysis of saphenous nerve in control WT and σ_1_R-KO mice

**Figure 2:**
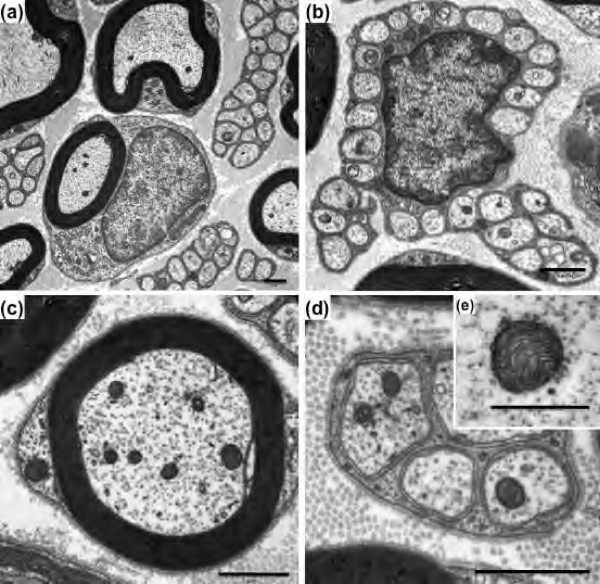
**Transmission electron micrographs of myelinated and unmyelinated fibers of the saphenous nerve from WT mice.** Cross-section of myelinated and unmyelinated fibers **(a)**; cross-section of unmyelinated fibers with Schwann cell nucleus **(b)**; high magnification of myelinated **(c)** and unmyelinated **(d)** fibers; representative photograph of a normal axonal mitochondrion **(e)**. Scale bar: 1 μm **(a-d)** and 0.5 μm **(e)**.

Figure 2 illustrates the ultrastructural characteristics of myelinated and unmyelinated fibers in WT control mice. Normal axoplasmic structures (neurotubules and neurofilaments) can be identified in both myelinated and unmyelinated fibers (Figure 2). Mitochondria within fibers are typically characterized as circular or oval structures containing cristae and amorphous electron dense material enveloped by double membranes (Figure 2e). The ultrastructural characteristics of myelinated and unmyelinated fibers in σ_1_R-KO control mice were similar to those of WT control mice. Hence, the results show an absence of significant histological differences between the different types of fibers in both groups of animals.

**Figure 3:**
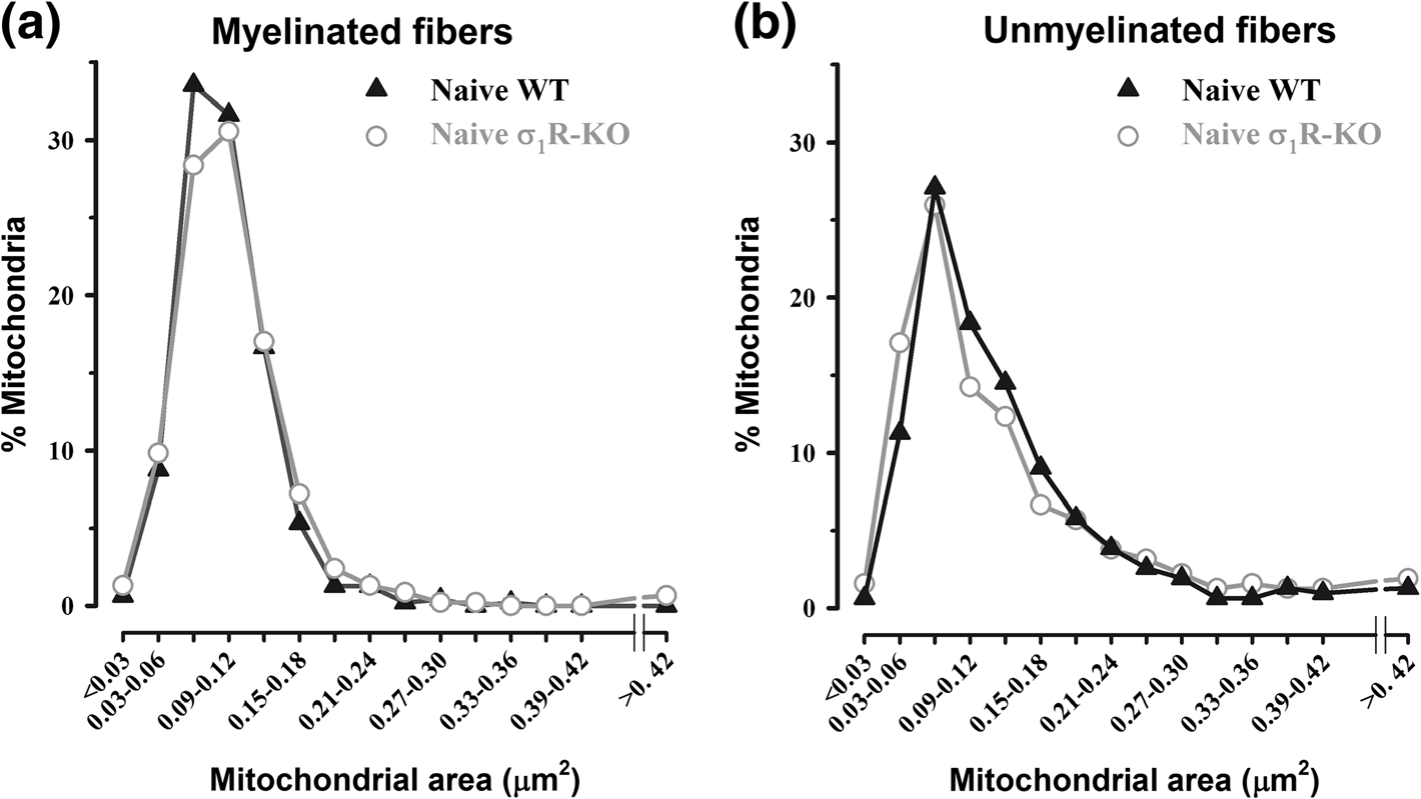
**Mitochondrial area distribution in fibers of saphenous nerve from WT and σ**_**1**_**R-KO mice.** Mitochondrial area distribution in myelinated **(a)** and unmyelinated **(b)** fibers of saphenous nerve from control WT and σ_1_R-KO mice. Morphometric analysis of the mitochondrial area showed a wide range of values (<0.03 to > 0.42 μm^2^) in both types of nerve fiber. Each point represents the percentage of mitochondria in a particular size range. Note the absence of differences between the mitochondrial area of WT and σ_1_R-KO animals in either fiber type.

Morphometric measurement of the mitochondrial area showed an ample range of values (< 0.03 to > 0.42 μm^2^) in the two types of saphenous nerve fiber, with a similar distribution pattern in WT and σ_1_R-KO control mice (Figure 3a and b). In fact, the mean areas of the mitochondria of each fiber type were virtually identical in both WT and σ_1_R-KO control mice (Table 1). However, in both groups of mice, the mean mitochondrial areas were significantly larger in unmyelinated C-fibers than in myelinated A-fibers (Table 1).

**Table 1:** **Comparison of mitochondrial area in myelinated and unmyelinated fibers of saphenous nerves obtained from naive wild type (WT) and σ**_**1**_**-receptor knockout (σ**_**1**_**R-KO) mice and from animals of both genotypes 10 days after treatment with paclitaxel or its vehicle**

		**Mean mitocondrial area (μm**^**2**^**)**	
**Genotype**	**Treatment**	**Myelinated**	**Unmyelinated**
**WT**	Naive	0.102 ± 0.002	0.135 ± 0.016^#^
	Vehicle	0.117 ± 0.008	0.164 ± 0.006^##^
	Paclitaxel	0.134 ± 0.007^**^	0.161 ± 0.009^#^
**σ**_**1**_**R-KO**	Naive	0.107 ± 0.005	0.134 ± 0.010^#^
	Vehicle	0.116 ± 0.005	0.164 ± 0.021^##^
	Paclitaxel	0.115 ± 0.006	0.170 ± 0.007^##^

Values represent the mean mitochondrial area of the results obtained in 3–5 animals per experimental group. Statistically significant differences in comparison to naive WT animals: ^**^P < 0.01 (one way ANOVA followed by Bonferroni test). Note that paclitaxel did not significantly enhance the mean mitochondrial area in σ_1_R-KO mice myelinated fibers, nor in any genotype in unmyelinated fibers. Statistically significant differences in comparison to myelinated fiber: ^#^P < 0.05; ^##^P < 0.01 (two way ANOVA followed by Bonferroni test). Note that mitochondrial area was greater in unmyelinated than myelinated fibers in all the experimental groups.

### Electron microscopy analysis of saphenous nerve fibers in paclitaxel-treated WT and σ_1_R-KO mice

**Figure 4:**
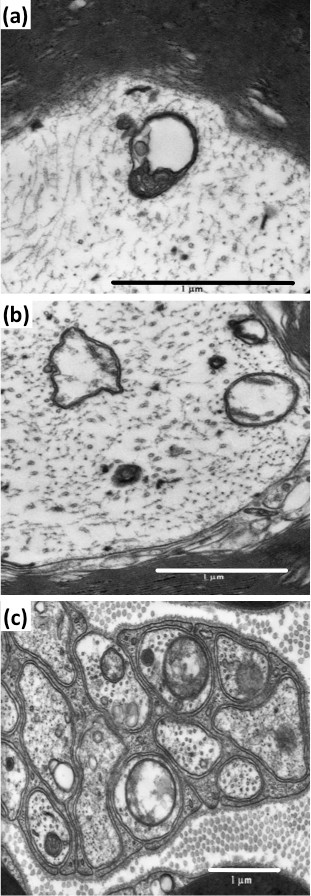
**Atypical axonal mitochondria in myelinated and unmyelinated fibers of saphenous nerve of paclitaxel-treated mice.** High magnification of a swollen mitochondrion with collapsed cristae at one pole, leaving a large vacuole **(a)**. Swelling and vacuolization, with loss of cristae and disorganized matrix, are prominent in several mitochondria in myelinated **(b)** and unmyelinated **(c)** fibers. Scale Bar: 1 μm.

Ultrastructural study of saphenous nerves from paclitaxel-treated WT and σ_1_R-KO mice showed no evidence of axonal, Schwann cell, or myelin degeneration, or of aggregates of neurotubules. However, mitochondrial alterations were observed in axons of saphenous nerves, especially in WT mice, at day 10 from the start of paclitaxel treatment. These alterations were associated with an enlarged size (swelling) and vacuolization of mitochondria (Figure 4), resulting in a statistically significant increase in mean mitochondrial area of 31% in A-fibers (from 0.102 to 0.134 μm^2^) of WT animals (Table 1). In contrast, paclitaxel-treatment only slightly (7%) and non-significantly increased the mean mitochondrial area in σ_1_R-KO mice (from 0.107 to 0.115 μm^2^) (Table 1). Paclitaxel also increased the mean mitochondrial area in unmyelinated fibers of WT and σ_1_R-KO mice, but this effect did not reach statistically significance in any genotype and was of similar magnitude than that produced by paclitaxel-vehicle (Table 1). Because previous studies [7,8] reported an increase in atypical mitochondria in paclitaxel-treated animals, we analyzed in detail the population of atypical mitochondria in the experimental groups.

**Figure 5:**
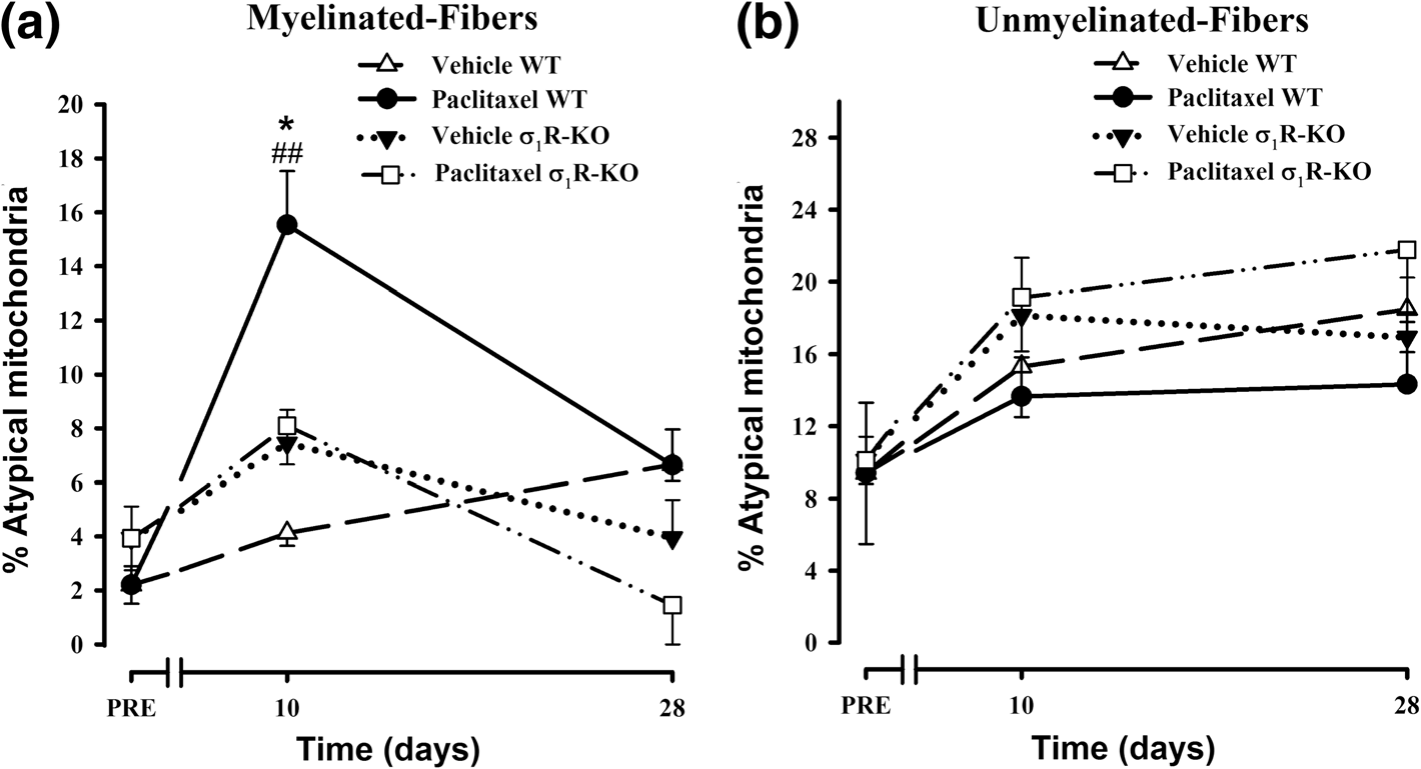
**Effect of the genetic inactivation of the σ**_**1**_**R on paclitaxel-induced mitochondrial abnormalities.** Effect of treatment with paclitaxel or its vehicle on the percentage of atypical mitochondria on myelinated **(a)** and unmyelinated **(b)** fibers of saphenous nerves from WT and σ_1_R-KO mice. Each point and vertical line represent the mean ± SEM of the percentage of atypical mitochondria relative to the total number of mitochondria in each type of fiber at day PRE (before treatment) and at days 10 and 28 posttreatment (n = 3-5 animals per day). Statistically significant differences between paclitaxel-treated WT mice and vehicle-treated WT mice and between the values on the pretreatment day and the days after treatment, ##*p* < 0.01; statistically significant differences between WT and σ_1_R-KO mice on the same day after treatment, *p < 0.05 (two-way ANOVA followed by Bonferroni test).

Figure 5 depicts the percentage of atypical mitochondria in the A- and C-fibers of WT and σ_1_R-KO mice before any treatment and at 10 and 28 days after the first dose of paclitaxel or its vehicle. Treatment with the paclitaxel-vehicle produced no statistically significant change in the percentage of atypical mitochondria in either fiber type in either WT or σ_1_R-KO mice (Figure 5a and b). However, at 10 days after the first administration of paclitaxel, a statistically significant and substantial increase in the percentage of atypical mitochondria was observed in the myelinated fibers of WT mice (from 2.20 ± 0.69% in pretreatment group to 15.53 ± 2.00% at day 10 posttreatment, a posttreatment:pretreatment ratio of 7.06) (Figure 5a). In contrast, the percentage of atypical mitochondria in the A-fibers of σ_1_R-KO mice showed a small and non-significant increase after 10 days of paclitaxel treatment (posttreatment/pretreatment ratio = 2.07) (Figure 5a). At day 10, paclitaxel-treated WT mice showed statistically significant differences with vehicle-treated WT mice and with both paclitaxel- and vehicle-treated σ_1_R-KO mice (Figure 5a). Analysis of the incidence of atypical mitochondria in C-fibers at 10 days of paclitaxel treatment revealed only small and non-significant increases in the percentage of atypical mitochondria in WT (posttreatment/pretreatment ratio = 1.45) and σ_1_R-KO (posttreatment/pretreatment ratio = 1.89) mice (Figure 5b).

**Figure 6:**
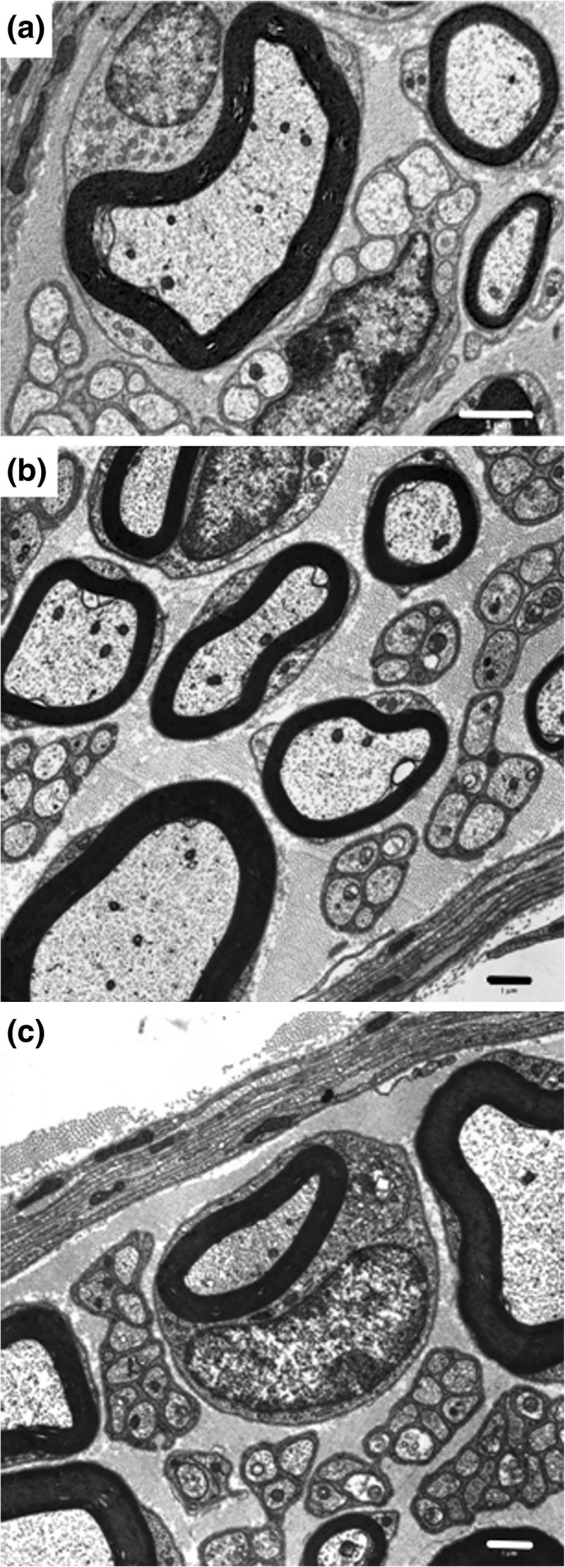
**Transmission electron micrographs of myelinated and unmyelinated fibers of the saphenous nerve 28 days after paclitaxel treatment.** Cross-section of myelinated and unmyelinated fibers of WT mice **(a)**, σ_1_R-KO mice **(b)** and BD-1063-treated WT mice **(c).** Scale bar: 1 μm.

At 28 days of paclitaxel treatment, no statistically significant changes were observed in the percentage of atypical mitochondria in myelinated or unmyelinated fibers in either WT or σ_1_R-KO mice (Figure 5a and b). At this day, the ultrastructural characteristics of myelinated and unmyelinated fibers and their mitochondria were undistinguishable from that observed in naive animals in both WT and σ_1_R-KO mice (Figure 6a and b).

### Electron microscopy analysis of saphenous nerve mitochondria in WT mice treated with paclitaxel and the σ_1_R antagonist BD-1063

**Figure 7:**
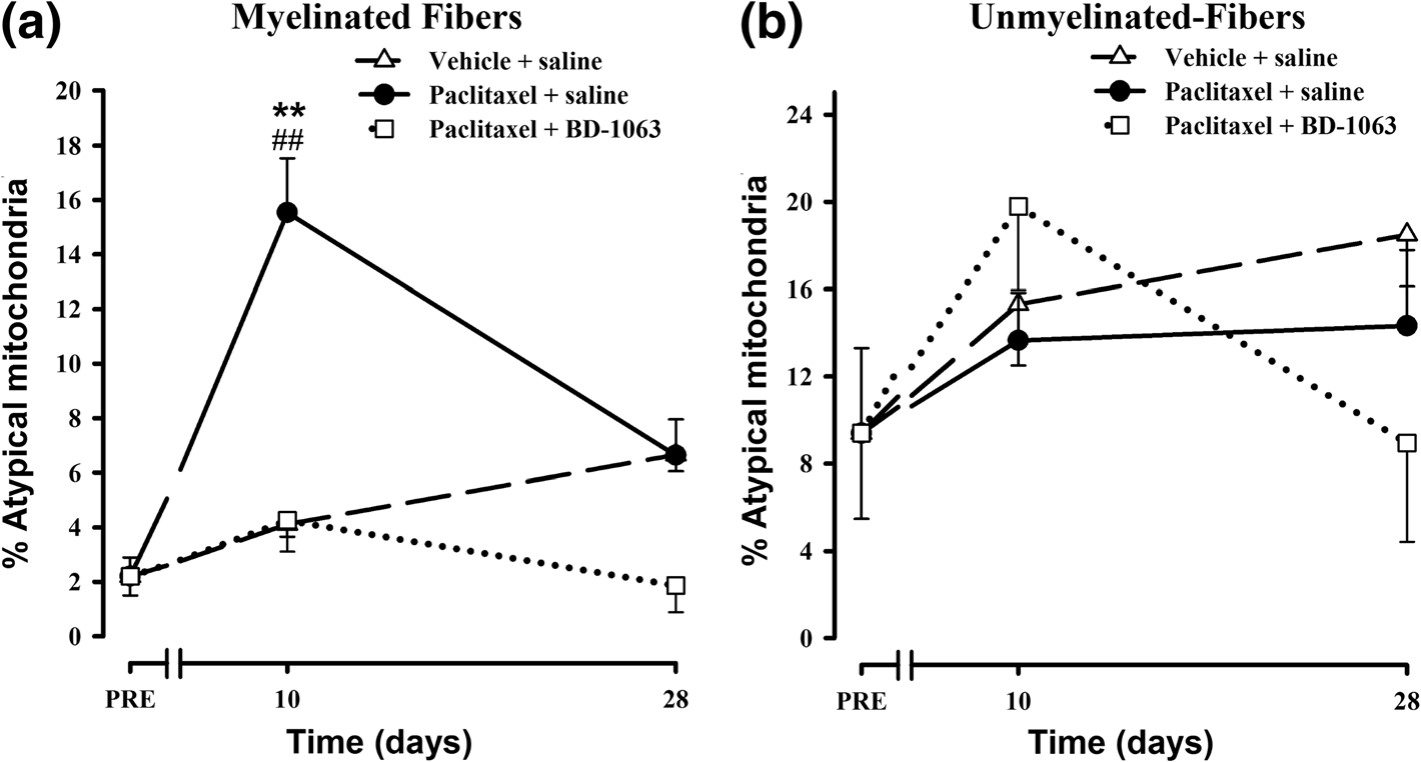
**Effect of the pharmacological blockade of the σ**_**1**_**R on paclitaxel-induced mitochondrial abnormalities.** Effect of treatment with paclitaxel + saline, paclitaxel-vehicle + saline, or paclitaxel + BD-1063 on the percentage of atypical mitochondria in myelinated **(a)** and unmyelinated **(b)** fibers of saphenous nerves from WT mice. Each point and vertical line represent the mean ± SEM of the percentage of atypical mitochondria relative to the total number of mitochondria in each type of fiber before treatment (PRE) and at days 10 and 28 posttreatment (n = 3-5 animals per day). Statistically significant differences between WT mice treated with paclitaxel + saline and the rest of the groups on the same day after treatment, **p < 0.01; and between the values on the pretreatment day and the days after treatment, ##*p* < 0.01 (two-way ANOVA followed by Bonferroni test).

Administration of BD-1063 (32 mg/kg, s.c.) before each paclitaxel dose completely prevented the paclitaxel-induced increase in the percentage of atypical mitochondria in myelinated fibers of WT mice at day 10 (Figure 7a). At day 28, the ultrastructural characteristics of saphenous nerve fibres (Figure 6c) and the percentage of atypical mitochondria in myelinated nerve fibers (Figure 7a) in BD-1063-treated animals were similar to those observed before paclitaxel treatment. Administration of BD-1063 did not produce any statistically significant effect on the percentage of atypical mitochondria in C-fibers (Figure 7b).

## Discussion

The main finding of this study is that the pharmacological blockade or genetic knockout of σ_1_R prevents the increased incidence of atypical axonal mitochondria in saphenous nerve myelinated fibers and the neuropathic pain signs associated with the administration of paclitaxel in mice. These findings suggest, for the first time, an involvement of the σ_1_R in the paclitaxel-evoked mitochondrial abnormalities that appear to be important in the pathophysiology of paclitaxel-induced neuropathy.

We confirm here that paclitaxel induces cold and mechanical allodynia in WT mice as previously reported [5,28,29]. However, when activation of σ_1_R was hindered, through a genetic or pharmacologic approach, the development of paclitaxel-induced allodynia was completely prevented, suggesting a key role for the σ_1_R in this type of neuropathic pain. These results are in agreement with those of previous studies demonstrating that σ_1_R-KO mice [23,24,30] and WT animals pretreated with σ_1_R antagonists [20-22,28,31] showed a marked reduction of pain in different models that activate central sensitization mechanisms. In addition, it has been reported that the spinal σ_1_R system contributes to diabetic neuropathic pain in mice [25]. Therefore, the present and previously published behavioral data strongly support the involvement of σ_1_R in modulating pain, especially neuropathic pain.

Our paclitaxel treatment schedule induced an increase in the frequency of atypical mitochondria in A-fibers of mouse saphenous nerve. These atypical mitochondria were always swollen and/or vacuolated (area > 0.20 μm^2^; diameter > 500 nm). These criteria are very similar to those used by authors who also found an increased incidence of atypical axonal mitochondria in peripheral nerves of rats with paclitaxel- [7,8], oxaliplatin- [32] or bortezomib-induced neuropathy [33]. An increase in swollen and/or vacuolated mitochondria has also been reported in the peripheral nerves [34] and DRGs [35] of animals with diabetic neuropathy and in the sural nerves of patients with painful peripheral neuropathy induced by 2’3’-dideoxycitidine (ddC) and HIV infection [36]. Hence, these mitochondrial structural alterations may be a common characteristic of these types of neuropathy.

Our finding of a low incidence of atypical mitochondria in naive mice appears to be a common observation in normal animals fixed with aldehydes [7,35]. In the present study, we processed all nerves using the same methodology; the mitochondria conserved their double membrane, their neighboring microtubules were well preserved, and the mitochondria from other cells (Schwann cells, fibroblasts, endothelial cells) were normal. Consequently, it is highly unlikely that the paclitaxel-induced increase in atypical mitochondria was due to an unsuitable fixation rather than to a neurotoxic effect of paclitaxel. We also found an increase, although not statistically significant, in atypical mitochondria in the saphenous nerves of mice treated with paclitaxel-vehicle, similar to that reported previously [8]. This is not surprising, given that one of its main components, Cremophor EL, can directly damage mitochondria [37].

It is known that paclitaxel-induced neuropathy is associated with a hypersensitization of A-fibers without affecting C-fibers in mice [4], and that paclitaxel mainly impairs the functionality of large myelinated (A-β) fibers in humans [1]. In agreement with these data, we found that paclitaxel induced a significant increase in atypical axonal mitochondria in A-fibers but not in C-fibers of saphenous nerves from WT mice. The time-course of the mitochondrial and behavioral alterations was similar, and both were evident on day 10 and resolved by day 28. An increase in axonal atypical mitochondria (also in parallel with the time-course of behavioral changes) was previously reported in paclitaxel-treated rats [7,8]. In these studies, however, both myelinated and unmyelinated fibers were affected and the behavioral and mitochondrial changes peaked later (27 days) than in the present study. The lack of significant effect of paclitaxel in unmyelinated fiber mitochondria of WT mice in our study could have been due to the greater percentage of atypical mitochondria in unmyelinated than myelinated fibers in control condition or to the greater variability in such a percentage in unmyelinated fibers (which would have affected the probability of reaching statistical significant differences). However, we do not think that this is the case since in WT-mice paclitaxel-treatment increases 7 times the percentage of atypical mitochondria in A-fibers but only 1.5 times in C-fibers. Because the percentage of atypical mitochondria in naive WT mice C-fibers is around 10% it would have been perfectly possible to increase this value also 7 times without reaching a plateau (since 90% of C-fibers mitochondria are typical in naive animals and therefore are susceptible to become atypical as a consequence of paclitaxel treatment) but, in fact, we do not observed such effect. Instead, differences between species may explain the discrepancy between C-fiber mitochondrial alterations induced by paclitaxel in rats [7,8] and in mice (present study), because important variations in the primary afferent unmyelinated neurochemistry between mice and rats have been previously described [38,39]. In fact, when similar protocols of paclitaxel treatment are used, the neuropathic pain induced by paclitaxel peaked earlier and it is of shorter duration in mice [5,29,40] than in rats [7,8]. Moreover, a different involvement of spinal cord microglial activation by paclitaxel in mice and rats has been reported [29,41,42]. Nevertheless, paclitaxel did not produce alterations in the mitochondria of Schwann cells in rats [43] nor in mice (present study). Although the details of the paclitaxel-induced neuropathy seems not to be the same in rats and mice, it is interesting to note that in both species paclitaxel induces qualitatively similar behavioral and mitochondrial changes, which suggest that these are core characteristics of paclitaxel neuropathy independently of the species considered. Therefore, our results in mice and those previously reported by Bennett’s group in rats suggest that paclitaxel-induced neuropathic pain may result from an impairment of axonal mitochondria. In fact, functional impairment of mitochondria was recently reported in peripheral nerves from paclitaxel- and oxaliplatin-treated rats [44].

Previous studies in mice [45-47] and rats [48-50] found evidence of axonal degeneration or alterations in Schwann cells or microtubules after paclitaxel administration. We did not observed any of these structural irregularities, probably because the single and cumulative doses used here were markedly below those administered in the above-mentioned studies (single dose, 2 mg/kg in the present study vs. 5–50 mg/kg in the others; cumulative dose, 10 vs. 20–280 mg/kg). This explanation is supported by the absence of these structural anomalies in other studies using similarly low doses [7,43] to those tested in the present study.

Genetic inactivation (σ_1_R-KO mice) or pharmacological blockade (σ_1_R antagonist) of the σ_1_R prevented paclitaxel-induced mitochondrial abnormalities and neuropathic pain signs. This suggests that the σ_1_R must be present and play a key functional role in the development of paclitaxel-induced painful neuropathy and atypical mitochondria. Therefore, the prophylactic effect of σ_1_R antagonists such as BD-1063 (present work; [28]) and S1RA [28] on the development of paclitaxel-induced cold and mechanical allodynia may be related to the prevention of these mitochondrial abnormalities. These data support the proposal of selective σ_1_R antagonists as a novel approach to the treatment of neuropathic pain [27].

It has been suggested that the mechanisms by which paclitaxel cause the mitochondrial abnormalities may derive from its binding to the β-tubulin associated with the voltage-dependent anion channel (VDAC) [44]. VDAC is the most abundant protein in the mitochondrial outer membrane [51] and, under certain situations (e.g., mitochondrial Ca^2+^ overload), may open the mitochondrial permeability transition pore (mPTP) and eventually produce mitochondrial alterations, including mitochondrial swelling and the release of accumulated Ca^2+^[52]. Thus, paclitaxel has been found to induce these effects *in vitro*[53-55]. Another possible explanation of paclitaxel-induced mitotoxicity is the indirect regulation of mPTP through the binding of paclitaxel to bcl-2, reversing the function of bcl-2 as a blocker of mPTP opening [56]. Taken together, these data suggest that paclitaxel may induce mPTP opening by binding to the β-tubulin joined to VDAC and/or to bcl-2, which would induce mitochondrial swelling and increase the release of mitochondrial Ca^2+^ to the cytoplasm. The σ_1_R modulates VDAC function [57] and tonically regulates the expression of bcl-2 proteins [58] and consequently may also indirectly regulate mPTP opening, inhibiting mitochondrial swelling and Ca^2+^ release. Further studies are warranted to test this hypothesis.

## Conclusions

In summary, we found that the pharmacological blockade (σ_1_R antagonists) or genetic inactivation of σ_1_R (knockout mice) prevents the sensory-nerve mitochondrial abnormalities induced by paclitaxel in parallel with the prevention of neuropathic pain development. These findings suggest that σ_1_R antagonists might have therapeutic value for the prevention of paclitaxel-induced neuropathic pain.

## Methods

### Animals

Experiments were performed in female WT (Charles River, Barcelona, Spain) and female σ_1_R-KO (Laboratorios Esteve, Barcelona, Spain) CD-1 mice weighing 25–30 g. The σ_1_R-KO mice were generated on a CD-1 background as previously described [23]. There are no gender difference in taxane-induced neuropathy [59], but since taxanes are frequently used in treatment of breast and ovarian cancer in women [60,61] we preferred to perform our experiments in female mice. The animals were housed in colony cages and kept in temperature- and light-controlled rooms (22 ± 1ºC, lights on at 08.00 h and off at 20.00 h, air replacement every 20 min). Testing took place during the light phase (from 9.00 h to 15.00 h). Mice were handled in accordance with the European Communities Council Directive of 24 November 1986 (86/609/ECC). The experimental protocol was approved by the University of Granada Research Ethics Committee.

### Drugs and drug administration

The drugs used were paclitaxel and the σ_1_ receptor antagonist BD-1063 (both from Tocris Cookson Ltd., Bristol, United Kingdom). Paclitaxel was dissolved in a solution of 50% Cremophor EL and 50% absolute ethanol to obtain a concentration of 6 mg/ml. This paclitaxel solution was diluted in sterile physiological saline to a final concentration of 2 mg/10 ml just before its administration. For control treatments, the paclitaxel-vehicle solution was also diluted just before its administration in saline at the same proportion as the paclitaxel solution. Paclitaxel (2 mg/kg) was administered intraperitoneally (i.p.) in a volume of 10 ml/kg once per day for 5 consecutive days (cumulative dose of 10 mg/kg); a schedule of paclitaxel treatment that produces a painful neuropathy in mice [5,28,29,40]. The control group was administered with the vehicle for paclitaxel according to the same schedule.

BD-1063 (32 mg/kg) was dissolved in saline just before the s.c. administration of a volume of 5 ml/kg in the interscapular area. This dose of BD-1063 produces a significant anti-allodynic effect in several models of pain [23,28,62]. The control animals received s.c. the same volume of saline. BD-1063 was s.c. administered to avoid any possibility of chemical interaction with paclitaxel solution (which was i.p. administered).

### General procedures for drug treatments and behavioral assays in pain models

The general procedures were performed as previously described [5,28] with slight modifications. First, behavioral responses were tested in each animal at 3 days before the start of paclitaxel administration (pretreatment value). Then, animals were treated with drugs once daily for 5 consecutive days. On each treatment day, animals received an s.c. injection of saline or BD-1063 and then, after a 30-min interval, an i.p. injection of paclitaxel or its vehicle. Post-treatment responses were measured on days 10 and 28 after the first paclitaxel or vehicle injection. These days were selected because the expression of paclitaxel-induced cold and mechanical allodynia is clearly established on day 10 [5,28] and there is no pain behavior on day 28 (Figure 1a and b). Each animal was tested alternately in both pain tests, with an interval of 24 h between evaluations, and was sacrificed after the final measurement in order to obtain the saphenous nerve, as described below. Only paclitaxel-treated WT animals that showed both cold and mechanical allodynia on day 10 were selected for the study of saphenous nerve ultrastructure. The criteria for considering that an animal had developed cold and mechanical allodynia were those previously described [5]: for cold allodynia, if the duration of acetone-induced licking or biting of the stimulated paws was higher or equal than 2 s; and for mechanical allodynia if the mean of the threshold values recorded on this day was lower (0.6 g or more) than the mean of the animal’s pretreatment values (obtained 3 days before paclitaxel treatment was started).

The experimenter who evaluated the behavioral responses was blinded to the treatment and genotype of experimental subjects. In all cases, experiments in the σ_1_R-KO or WT groups, vehicle- or paclitaxel-treated groups, and saline- or BD-1063-treated groups were run in parallel.

### Procedure to measure cold allodynia

Cold allodynia was tested by gently touching the plantar skin of the hind paws with an acetone bubble using a syringe connected to a thin tube as previously described [5,28]. On each evaluation day, the mice were habituated for 30 min in individual transparent boxes (7 × 7 × 13 cm) on an elevated platform with a wire mesh floor. After the adaptation period, acetone was applied alternately three times to each hind paw at intervals of 30 s, and the duration of licking or biting was recorded with a stopwatch and reported as the cumulative time of licking/biting at all six measurements. A cut-off time of 10 s was used in each of the six trials.

### Procedure to measure mechanical allodynia

Mechanical allodynia was assessed by measuring the threshold force for hind paw withdrawal with an electronic Voy Frey apparatus (Dynamic Plantar Aesthesiometer, Ugo Basile, Comerio, Italy) as previously described [5,28]. This electronic device uses a single nonflexible filament that applies increasing force (from 0 to 10 g) against the plantar surface of the hind paw over a 20-s period. The nocifensive paw withdrawal response automatically turns off the stimulus, and the mechanical pressure that evokes the response is recorded. On each day of the experiment, the mice were habituated for 2 h in individual transparent boxes (9 × 9 × 14 cm) with a wire mesh bottom and then tested three times alternately in each hind paw, allowing at least 30 s between each measurement. The mean of the six trial values was considered the response of the animal.

### Procedure to obtain saphenous nerves and electron microscopy analysis

Mice were anesthetized with isofluorane (IsoVet®, B. Braun, Barcelona, Spain) and perfused intracardially with 20 ml saline followed by 30 ml of freshly prepared 2% glutaraldehyde/1% paraformaldehyde in 0.1 M phosphate buffer (PB), pH 7.4, for 15 min. After perfusion, saphenous nerves were dissected and processed as previously described by Flatters and Bennett [7] with slight modifications. Briefly, 5 mm of saphenous nerves were dissected at mid-thigh level and fixed with 2% glutaraldehyde/1% paraformaldehyde in 0.1 M PB, pH 7.4, overnight at 4°C. After fixation, samples were transferred to 10% sucrose in 0.1 M PB for 24 h at 4°C and then postfixed with 0.1% osmium tetroxide in 0.1 M PB, pH 7.4, containing 1% potassium ferrocyanure for 1 h at 4ºC, dehydrated in a graded series of alcohols, and embedded in Epoxy resin. Samples were sectioned with an Ultracut E Reichert-Jung ultramicrotome (Leica, Barcelona, Spain) to obtain ultrathin sections (70 nm) and then stained with uranyl acetate and lead citrate.

Ultrathin sections were viewed in a Zeiss EM 902 (Zeiss, Oberckochem, Germany) transmission electron microscope equipped with a monochrome CCD camera. Micrographs were taken with the camera connected to a video frame grabber (Snappy Video Snapshot, Play Inc., Rancho Cordova, CA) plugged into a PC (1500x1125 resolution). To perform ultrastructural and morphometric analyses, microphotographs were taken of myelinated (n = 30) and unmyelinated (n = 30) axons of the saphenous nerves from each mouse. ImageJ software (http://rsb.info.nih.gov/ij/index.html) was used to measure the area (A), perimeter (P), circularity (4π[A/P^2^]; 1.0 = perfect circle), and Feret’s diameter (longest distance between any two points along the selection boundary). Nerve fibers were classified according to the presence or absence of the myelin sheath as myelinated (A-fiber, n = 2100) or unmyelinated (C-fiber, n = 2100) fibers. Morphometric measurements were conducted at 20000× for the area, perimeter, circularity, and Feret’s diameter in mitochondria from A- and C-fibers. Area values alone are given in the Results section for the mitochondria, because the other morphometric data were closely related to the area and were similarly affected by treatments (data not shown). Morphometric analyses were performed by observers who were blind to the genotype or treatment group of the mice.

### Analysis of mitochondria

Mitochondria from myelinated and unmyelinated fibers were identified as circular or oval structures with a double membrane containing cristae and amorphous electron dense material that had an area of at least 0.02 μm^2^ (diameter > 165 nm). Analysis of mitochondrial area (n = 10679) in the different fiber types in both WT and σ_1_R-KO control mice allowed the observation of mitochondrial populations of different sizes (see Results section). Atypical mitochondria were observed in both A- and C-fibers (see Results section) and defined on the basis of a double membrane, vacuolization and/or pronounced swelling, according to the criteria described by Flatters and Bennett [7], slightly modified. Thus, pronounced swelling was defined as an enlargement of at least 2-fold the mean mitochondrial area of naive mice. Given that the mitochondrial area was significantly larger in the C-fibers than in the A-fibers of both WT and σ_1_R-KO control animals (see Table 1), a pronounced swelling of a mitochondrion was considered when a mitochondrion had > 0.20 μm^2^ (diameter > 500 nm) in the myelinated fibers and > 0.26 μm^2^ (diameter > 580 nm) in the unmyelinated fibers. Vacuolated mitochondria are frequently characterized by the accumulation of electron-dense material in one of the poles of the mitochondria. The incidence of atypical mitochondria was expressed as the percentage of atypical mitochondria relative to the total number of mitochondria measured.

### Data analysis

Differences between values in the behavioral assays were analyzed with two-way repeated measures analysis of variance (ANOVA) followed by the Bonferroni test. Differences between the frequencies of atypical mitochondria were analyzed with two-way analysis of variance (ANOVA) followed by the Bonferroni test. Differences between the mean mitochondrial area were analyzed with one-way and two-way analysis of variance (ANOVA) followed by the Bonferroni test. SigmaPlot 12.0 (Systat Software Inc., San Jose, CA) was used for all data analyses. Differences between means were considered statistically significant when the value of *p* was below 0.05.

## Abbreviations

σ1R: Sigma-1 receptor; σ1R-KO: Sigma-1 receptor knockout; WT: Wild-type.

## Competing interests

The authors declare that they have no competing interests.

## Authors’ contributions

FRN, CMC, FJC and MAC performed experiments, analyzed data and wrote the manuscript. JMV, EFS and JMB designed, coordinated and supervised the experiments as well as wrote the manuscript. All authors discussed the results, commented on the manuscript and approved the final version of the manuscript.
